# E‐Learning in Dental Medicine: A Key to More Equality?

**DOI:** 10.1111/eje.13080

**Published:** 2025-02-18

**Authors:** Amely Hartmann, Sara Steinhoff, Bilal Al‐Nawas, Diana Heimes, Peer W. Kämmerer

**Affiliations:** ^1^ Clinic and Polyclinic for Oral and Maxillofacial Surgery—Plastic Surgery University Medical Center Mainz Mainz Germany

**Keywords:** Covid‐19, dentistry, gender equality, remote learning, TKS‐WLB, work‐life balance

## Abstract

**Introduction:**

The COVID‐19 pandemic prompted a rapid shift towards remote learning in dental education, influencing practicing dentists' work‐life balance (WLB). This German nationwide questionnaire study, conducted in September 2022, aimed to explore the impact of transitioning to remote learning on the WLB of dentists, considering factors such as gender, parental status and professional roles.

**Methods:**

A voluntary and anonymous online survey involving 672 dentists utilised the ‘Trier Short Scale for Measuring Work‐Life Balance’ (TKS‐WLB). Statistical analyses used *R*, employing descriptive statistics for metric variables and mixed models for group comparisons. Post hoc analyses utilised *t*‐tests.

**Results:**

Overall, participants reported an enhanced WLB post‐transition. Gender disparities were observed, with men consistently experiencing better balance than women. The primary motivator for online training participation was the reduction of travel‐related challenges, cited by 92% of respondents. Notably, 52.8% faced no issues with the transition, and 55.5% found online training to complement in‐person events effectively. Women (71.8%) and participants with children (73.2%) expressed a preference for future asynchronous online training opportunities.

**Conclusions:**

This study underscores the positive impact of remote learning on the WLB of dentists, extending beyond the pandemic's challenges. Innovative online training programs, particularly beneficial for women and those with family commitments, have the potential to reshape dental education.

## 1 Introduction

The global surge in severe acute respiratory infections due to the SARS‐CoV‐2 virus has significantly impacted various sectors, including dentistry. Recognising the gravity of the situation, the World Health Organisation (WHO) declared the outbreak a pandemic in March 2020. Numerous countries swiftly implemented physical distancing measures to mitigate the spread of infections [1]. For dentistry, these unprecedented circumstances posed significant challenges to traditional training models and ongoing education [2]. The necessity for physical distancing and concerns about infection control prompted a paradigm shift in the approach to dental education. In‐person workshops, conferences and hands‐on training sessions, integral to continuous professional development, faced disruptions. Dental professionals and educators swiftly adapted to the evolving situation by embracing virtual platforms for training and education. Webinars, online courses and virtual conferences became the new norm, providing an alternative avenue for knowledge dissemination [2, 3]. This transition ensured the safety of practitioners and educators and demonstrated the resilience and adaptability of the dental community [4, 5]. Integrating technology into dental education offered unique interactive and engaging learning experiences. Virtual platforms facilitated global collaboration, allowing dental professionals to connect, share insights and stay abreast of the latest developments in the field. Additionally, online resources and e‐learning modules provided flexibility, enabling practitioners to engage in continuous education while navigating the challenges posed by the pandemic [2, 6].

Adopting remote training for dentists, while offering increased flexibility, has led to heightened stress levels, particularly impacting families with children and women in both professional and personal spheres. The blending of work and home life within remote settings has intensified stressors, especially for parents navigating dual responsibilities. Historically shouldering domestic burdens, women face additional challenges in managing professional commitments and increased caregiving responsibilities, emphasising the need for targeted support systems [7, 8].

Though widely used, the term ‘work‐life balance’ (WLB) lacks a singular definition and encompasses various dimensions that delve into the intricate interplay between professional and personal life. From a scientific standpoint, it entails exploring the multifaceted dynamics shaping the relationship between work and private life. Understanding WLB goes beyond a one‐size‐fits‐all approach, acknowledging the complexity of factors influencing individuals' experiences in both professional and personal domains [9]. The perception of WLB frequently varies between genders. For women, WLB is often framed within the context of ‘reconciling family and work,’ emphasising the challenges of balancing familial responsibilities with professional obligations. In contrast, discussions about WLB for men tend to prioritise ‘burn‐out,’ overall health considerations, and the significance of creative breaks [10]. This gendered lens highlights distinct perspectives and challenges associated with achieving a harmonious balance between work and personal life. The increasing employment and qualifications of women in recent decades have led to shifts in both the distribution of family and domestic tasks and the portrayal of role models within the professional world. These changes reflect evolving societal norms and contribute to a more diverse and equitable representation of women across various spheres of life. In this context, it becomes evident that both men and women attribute equal importance to work and private life, leading to minimal differences in personal career goals for individuals with comparable qualifications. This highlights the growing recognition of the significance of achieving a harmonious balance between professional and personal aspirations, irrespective of gender [11]. Nevertheless, when it comes to future life plans, women more frequently express a desire to attain WLB. This emphasises the evolving perspectives and priorities regarding career and personal life, with an increasing focus on achieving a harmonious equilibrium. Consequently, discernible gender differences may emerge in the so‐called ‘longer‐term balancing process,’ influencing the trajectories of professional careers for both men and women. This has prompted a growing emphasis on empirical research addressing the challenge of reconciling family and career, recognising the evolving dynamics of WLB in contemporary professional landscapes [12].

In recent years, the concept of WLB in dentistry has become closely linked to the rising proportion of women in the profession. Dentistry is experiencing a transformative shift, with discussions framing it as a ‘feminization’ of the dental field [13, 14]. The increasing presence of women in dentistry is poised to not only transform the profession itself but also, to some extent, reshape the structures within dental practices [15]. This transformation is apparent in shifting expectations within the dental profession, the interplay between work and family, and variations in start‐up, treatment and specialisation behaviours observed among female dentists compared to their counterparts [13]. Studies from New Zealand, Australia and England have demonstrated that female dentists are notably more inclined to work part‐time due to household and childcare responsibilities, leading to more frequent career breaks than their male counterparts [16]. This pattern leads to women perceiving their careers as significantly more affected than men's, highlighting the importance of addressing gender‐specific challenges in the dental profession [17].

The primary objective of this study was to explore the potential impact of e‐learning on enhancing the WLB of practicing dentists, with a specific focus on addressing challenges encountered by dentists with family responsibilities.

## 2 Material and Methods

### 2.1 Online Survey

Between September and October 2022, a voluntary and anonymous online survey was conducted involving 672 dentists from Germany (male = 227; female = 444; divers = 1). Ethical approval was obtained from the local ethics committee. The questionnaire, developed using the ‘LimeSurvey’ portal, was distributed through social networks such as Facebook, Instagram, WhatsApp and newsletters from the Lower Saxony Chamber of Dentists and the professional association ‘Dentista e.V.’. Alongside sociodemographic inquiries about age, gender and parental and professional status, participants were asked to assess past online training experiences. Evaluation criteria included satisfaction, learning effectiveness, logistical challenges and preferences for future training opportunities, utilising multiple‐choice questions and 6‐point Likert scales. The ‘Trier Short Scale for Measuring Work‐Life Balance’ (TKS‐WLB) [18] was employed for participants to rate their WLB before and after the shift to ‘remote learning’ prompted by the pandemic. The study complied with the STROBE protocol.

### 2.2 Statistical Analysis

Data analyses were conducted using R's statistical software packages, including lavaan, lme4, lmerTest, MuMIn and semTools [10, 19, 20, 21]. Only fully completed questionnaires were included in the analysis. Descriptive analyses were employed to calculate the mean and standard deviation (M[SD]) for metric variables, and absolute and relative frequency (n(%)) for categorical variables. To evaluate the reliability of the TKS‐WLB, single‐factor models were constructed using confirmatory factor analysis. The internal consistency of the models was assessed using McDonald's omega.

Acceptable reliability was established with values of 0.70 and above. A comparison between groups was conducted through mixed models with random intercepts. Q‐Q plots were utilised to assess the normal distribution assumption for the dependent variable. In the model, predictors were represented by the variable time (t) as a within‐subject factor and sociodemographic variables as between‐subject factors. Effect size was determined by the variance clarification *R* ^2^, with standardised cut‐off values of 0.01 indicating a small effect, 0.06 for a medium effect and 0.14 for a large effect. This rigorous approach allowed for a nuanced understanding of the impact of e‐learning on the WLB of practicing dentists [22]. Subsequent post hoc comparisons were conducted using *t*‐tests, and effect sizes were measured using Cohen's *d*. The standardised cut‐off values for interpreting the effect sizes were 0.20 for a small effect, 0.50 for a medium effect, and 0.80 for a large effect [23]. To address the issue of multiple testing in the ANOVA, a significance level of *p* < 0.05 was initially applied. However, considering the number of ANOVAs conducted for the dependent variables of gender, parental and occupational status, a correction was implemented. The adjusted significance level for our study was set at *p* < 0.017.

## 3 Results

The study encompasses a sample of *n* = 672 individuals actively engaged in ongoing online education within the dental field, either presently or within the last 2 years.

To assess the reliability of the WLB scale (TKS‐WLB), McDonald's omega for internal consistency was calculated, resulting in high values of t1 = 0.910 and t2 = 0.902, indicating strong internal consistency across both measurements. In the analysis of variance, notable changes between t1 and t2 are observed, particularly in the categories of time and gender, suggesting an overall enhancement in WLB over time (t1: 19.18 [5.81] vs. t2: 20.13 [5.59], *p* < 0.001, *R* ^2^ = 0.007) (Table 1). Men consistently demonstrated superior WLB (20.81 [5.83]) compared to women (19.08 [5.57]), irrespective of the time of measurement (men vs. women *p* < 0.001, *R* ^2^ = 0.020). A *p*‐value of 0.037 indicates a minor effect favouring individuals without children (20.11 [6.02]) over those with children (19.24 [5.41]) (Table 1). The occupational status of the participants, whether employed or self‐employed, did not yield significant results in the primary analysis of variance.

**TABLE 1:** Analysis of variance for work‐life‐balance (as a function of time, gender, parental or professional status).

Variable	Effect	*F*	*p*	*R* ^2^
Time	**Time**	42.73	**< 0.001**	0.007
Gender	Time	37.08	**< 0.001**	0.007
	**Gender**	15.86	**< 0.001**	0.020
	Time × gender	0.08	0.775	0.027
Children/no children	Time	42.27	**< 0.001**	0.007
	**Children/no children**	4.38	**0.037**	0.006
	Time × children/no children	0.71	0.711	0.013
Self‐employed/employed	Time	40.55	**< 0.001**	0.007
	Self‐employed/employed	0.17	0.678	0.000
	Time × self‐employed/employed	0.43	0.514	0.007

*Note:* Bold values definied as significant *P* < 0.05.

96.9% of the participants engaged in online dental education over the past 2 years, with the primary motivation being the elimination of lengthy travel distances for 92% of them. Additionally, 86.9% valued the flexibility in terms of time and location. However, challenges such as the lack of personal exchange and practical exercises were identified as primary reasons against online training. Participants were further prompted to assess their overall satisfaction with online programs, individual learning effectiveness and logistical and time‐related challenges using a 6‐point Likert scale [32].

**FIGURE 1:**
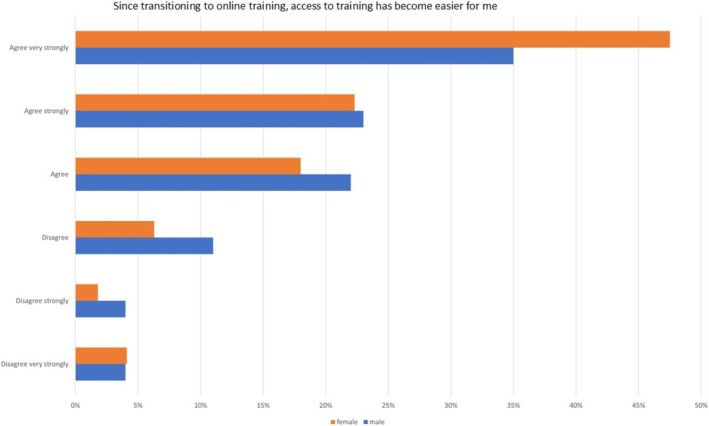
The 6‐point Likert scale showing results for the question ‘since transitioning to online training access to training has become easier for me’ based on gender.

**FIGURE 2:**
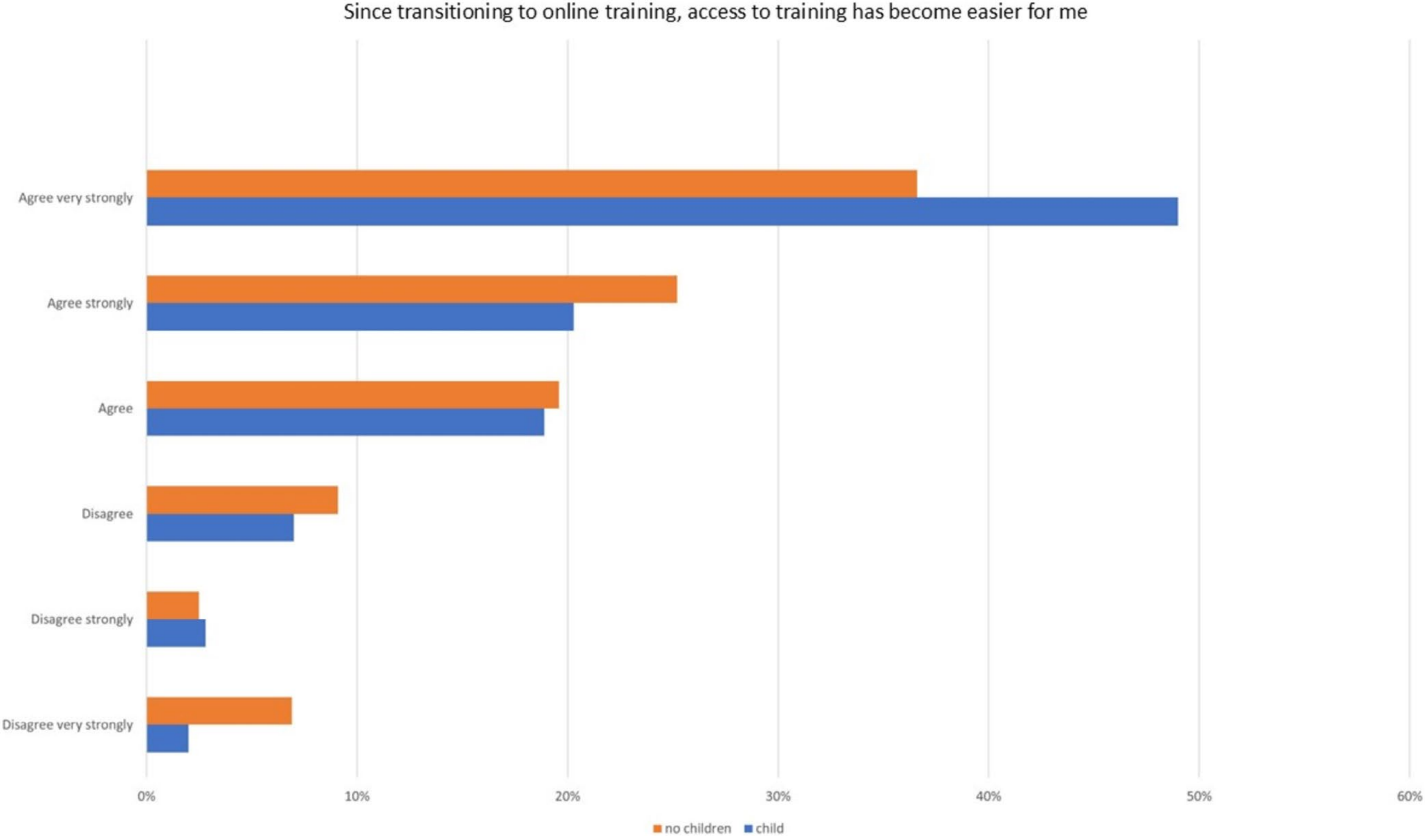
The 6‐point Likert scale showing results for the question ‘since transitioning to online training access to training has become easier for me’ based on parental status.

Analysing the responses based on gender (Figure 1) and parental status (Figure 2), it was observed that 60.6% of women considered online training an effective complement to in‐person events. Additionally, 47.5% of female respondents found online training a more convenient option for further training, compared to 34.8% of male respondents. Gender‐based differences in endorsing online formats for postgraduate training opportunities, such as structured training courses or postgraduate Master's programs, were notable. In 41.2% of cases, female dentists expressed a preference for online options, whereas only 26.9% of male dentists shared this inclination.

Similarly, 49% of dentists with children found accessing training through the online format notably easier, in contrast to 36.6% of dentists without children. The transition was generally perceived as easy by childless dentists (50.5%) and those with children (55.2%). However, a major difference was observed when considering interruptions or distractions within the work environment. 47% of dentists without children could follow their training courses without interruptions, while this proportion was significantly lower for dentists with children at 33.8%.

Both dentists without children (52.1%) and dentists with children (58.6%) found online training to be a valuable complement to in‐person events, with the latter group showing a slightly higher percentage. Digital postgraduate training notably received more approval from participants with children (40.3%) than those without children (31.9%), reflecting a preference dependent on their child status.

**FIGURE 3:**
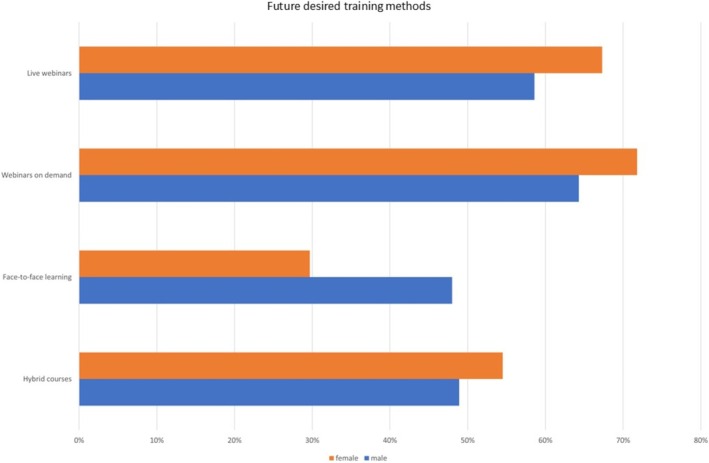
Desired training methods based on gender.

**FIGURE 4:**
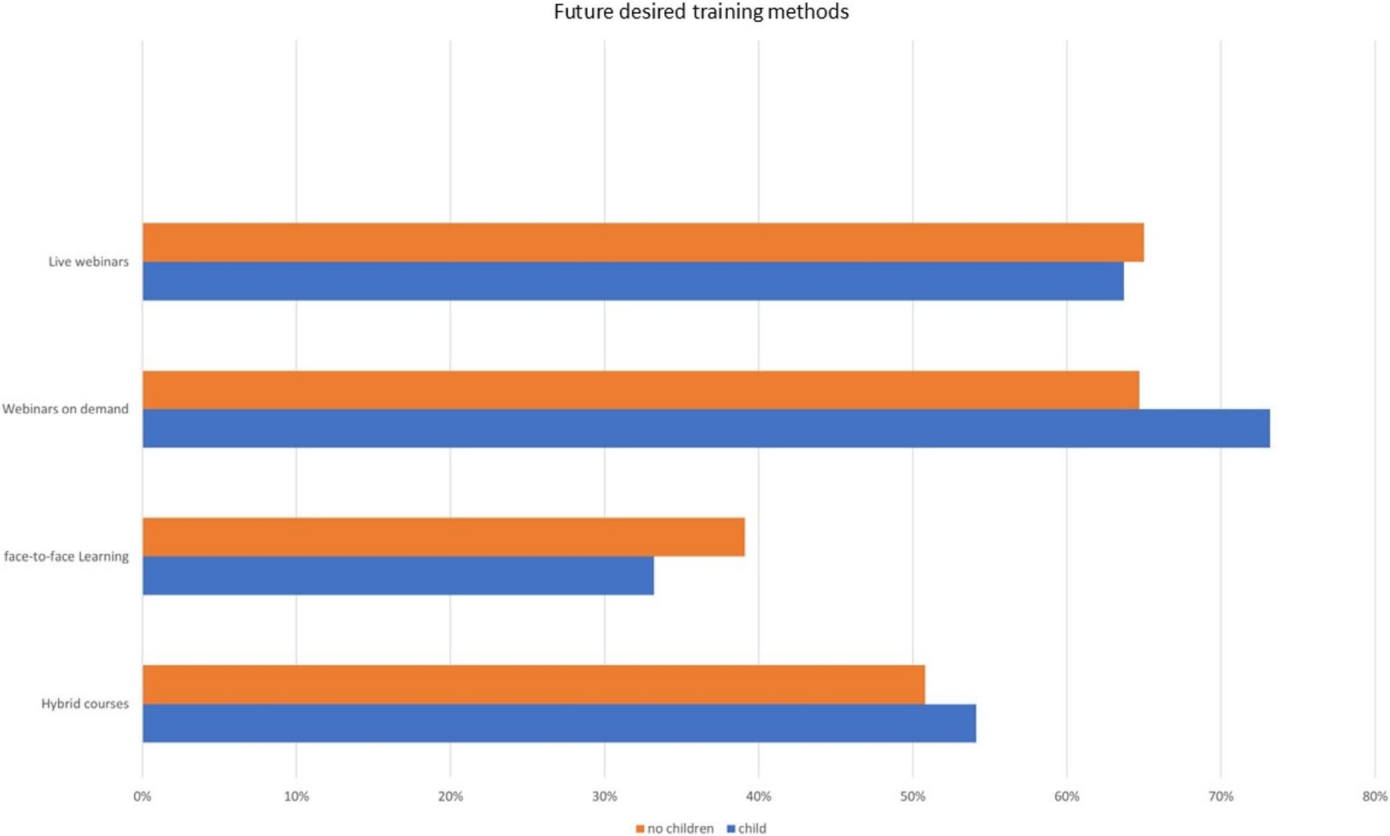
Desired training methods based child status.

Finally, the participants were asked what training courses they would prefer (Figures 3 and 4). The questionnaire presented options: in‐person events, hybrid courses and asynchronous and synchronous online courses. Participants were able to make multiple entries. Gender and child status were considered, recognising the assumption that women and dentists with family responsibilities may express preferences for training formats, ensuring problem‐free and efficient participation compared to their colleagues without children.

The most significant differences emerged for asynchronous webinars. 71.8% of female dentists and 73.2% of dentists with children preferred this format in the future, compared to 64.3% of male dentists. Another notable difference was the desire for a return to classroom‐only courses: 48% of male dentists preferred classroom‐based courses, compared to only 29.7% of female dentists. Hybrid courses were found to be a preference for half of the respondents in both groups (men 48.9%, women 54.5%, with children 54.1%, childless 50.8%).

## 4 Discussion

Despite external impacts, such as delivery shortages, emergency treatments and social isolation triggered by the COVID‐19 pandemic [9], our study revealed a significant improvement in the perceived WLB of all participants over time. However, it remains uncertain whether this can be solely attributed to the introduction of online training [18, 24].

The gender‐based differences observed in WLB raise important considerations. Women consistently reported lower WLB than men, a finding consistent with existing literature on gender disparities in WLB. The reasons behind these disparities are likely multifaceted and may include traditional gender roles, expectations and societal norms. The pandemic may have exacerbated these gender differences, especially with the added responsibilities associated with childcare and household duties. This underscores the necessity for proactive measures aimed at fostering a balanced WLB, especially for women. Furthermore, the findings suggested a slight, though statistically insignificant, advantage in WLB for participants without children in comparison to those with children.

The motivations for engaging in online training, such as eliminating lengthy travel distances and providing flexibility in time and location, align with the benefits often associated with remote learning [25]. Four main aspects contributed to the improved WLB such as flexibility, no need to commute, opportunity for asynchronous learning and access to specialisation. Online learning platforms offer the opportunity to complete courses and materials according to an individual schedule, which is particularly valuable for people with family commitments. Eliminating travel time saves valuable hours that can be used for family or other responsibilities. Asynchronous learning provides the ability to learn without any pressure or fixed time. With the global availability of online courses, dentists can choose programs suited to their needs without relocating or travelling for extended periods of time. The high preference for asynchronous webinars, especially among women and those with children, indicates a desire for flexibility and self‐paced learning. Concerning factors contributing to time management challenges in online continuing education, self‐discipline seems to be the main topic. Without fixed schedules, motivation can be difficult (less relevant in this study, but a factor in online learning in general). Distractions at home like household chores or family commitments can impair concentration. Technical problems, specifically difficulties with the internet connection or the platform can interrupt the learning process. Finally, the lack of direct exchange may play a role in the learning process. The need to clarify queries by email or forum can take up more time than face‐to‐face discussions.

When interpreting the data within the German context, variations in WLB between fathers and mothers appear to be frequently, though not exclusively, associated with work‐related factors such as working hours or the quality of work (including physical and mental stress, income adequacy and job security). The challenges associated with interruptions or distractions during online courses, particularly for dentists with children, emphasise the need for targeted support or accommodations. The impact of occupational status on WLB seems to be nuanced, and it cannot be presumed that WLB is inherently ‘better’ among salaried employees or self‐employed individuals. There is a societal notion that women generally choose to work as employees rather than establishing their own practices due to a perceived alignment of family and career, thereby achieving a supposedly superior WLB [26, 27]. Though, neither the present study nor the existing literature confirm this offering no uniform consensus in regard of this question. However, promoting flexible work approaches could provide an advantage, especially for female freelancers.

Practicing female dentists found online training courses to be a valuable complement to in‐person events. Additionally, they perceived it as a more convenient option to access further training within their everyday professional lives. It would have been insightful to explore whether the compatibility of family and career is related to easier online training access for women. However, as this aspect was not explicitly addressed, any potential connection remains speculative [24].

Participation in future postgraduate training, such as curriculums or Master's degree programs, appears preferable for female dentists if these were mostly provided online. 48% of male dentists preferred a return to exclusively in‐person teaching, in contrast to only 29.7% of female dentists. The majority of women, 71.8%, expressed a preference for pre‐recorded ‘on‐demand’ webinars. These findings provide a crucial foundation for the strategic planning of future training initiatives, as already implemented by the ‘Committee for the Interests of Female Dentists’. The committee is dedicated to developing targeted training measures to facilitate the swift integration of female dentists into specialised areas of dentistry, for example, after periods of absence such as pregnancy. Re‐entering the workforce after pregnancy or parental leave poses challenges for many female dentists, as they may lack practical experience in their daily practice. While online training courses cannot fully substitute for hands‐on experience, they provide the opportunity to expand knowledge and pursue specialisation, even during career breaks. Similarly, men facing the decision of ‘career or child’ may achieve a more balanced approach through flexible options for further training and specialisation, enabling them to take parental leave or reduce their working hours. By doing so, men can navigate these decisions without automatically facing stagnation on the career ladder [28].

The anonymous online questionnaire utilised in this study was crafted and validated, employing established and scientifically validated methodologies. To ensure the robust comparability and evaluability of the collected data, the questionnaire was meticulously designed, comprising solely of Likert scales and multiple‐choice questions. The Trier Short Scale for measuring perceived Work‐Life Balance (TKS‐WLB) was integrated twice in our questionnaire, presented in a randomised order and against different backgrounds. The TKS‐WLB scale was selected because it captures the multidimensional nature of work‐life balance, including gender and family nuances. It offers indicators of family responsibilities like reports about stress in general and conflicts arising from caregiving responsibilities. The scale also provides the opportunity to consider gender differences. It includes potential role conflicts that can be exacerbated by traditional gender roles. Concerning ethical aspects participation was anonymous and voluntary. The participants were informed in advance about the objectives and procedure. Personal data such as age and number of children were requested in ranges ‘from‐to’ so that no assignment to real persons could be made. The study was supervised by the Data Protection Officer (DPO) at the University of Mainz.

Perceived WLB is inherently subjective, shaped by individual assessments of various factors [24]. Direct quantification of WLB is challenging and requires operationalization, as outlined in the introduction. Participants faced the additional challenge of evaluating their WLB retrospectively, considering the period before the transition to online training, influenced in part by the ‘fading affect bias’ [29]. Ideally, assessments of TKS‐WLB should not have been confined to a questionnaire. However, the unpredictable nature of the COVID‐19 pandemic and the sudden shifts in dental training allowed little room for an alternative study design.

Recruitment via social media and newsletters was specifically aimed at reaching a large and diverse group of participants. However, this might comprise potential bias. People who are actively involved in social networks and newsletters could have specific demographic or professional characteristics that might have influenced the results. Conversely, people who do not regularly use online media may have been excluded, which limits the generalizability of the results (limited target group).

We recognise this limitation and plan to address this issue in future studies, recruitment should be supplemented by alternative methods (like personal approach or random sampling) in order to achieve more representative results.

Accrediting bodies should be integrated in regulating online continuing education by providing quality assurance and scientific content. It is also necessary to define standards for learning platforms and examinations in order to ensure comparability. Regular audits and assessments of providers can ensure the integrity and effectiveness of programs. Accrediting bodies could also promote innovative learning approaches and technologies tailored to the needs of dentists.

Future research should investigate the long‐term impact of online learning on clinical skills, patient care and professional satisfaction. Comparative studies could aim to analyse face‐to‐face and online training to compare their effectiveness. A more focused view could be given to different target groups and the need to adapt online learning for dentists with different experiences, specialties or family commitments. Technological advances might be taken into count like investigating the potential of new technologies such as virtual reality or artificial intelligence for continuing dental education. To optimise online learning platforms in the future, they should be fully mobile compatible so that courses can be accessed from anywhere. Concerning content, it should be available in short modules that can be easily integrated into everyday life (modular courses). Access to mentors or real‐time technical support can solve challenges faster. Community features in terms of forums/groups where dentists can share experiences and questions promote social exchange and motivation. Simple navigation and clear instructions help to save time and minimise distractions. Dental professionals could effectively integrate new technologies by a combination of face‐to‐face and online teaching indicating to theory online, practice on site. Theory should be taught online through videos or specific education material, while face‐to‐face teaching is used for discussions, questions and practical exercises. This provides the advantages of traditional teaching and the chances of the digital world. It is ideal for meeting the needs of modern learners, but requires careful planning and support to realise its full potential.

## 5 Conclusion

The present findings suggest that practicing dentists, especially those with family responsibilities, positively perceived the shift to online training. This implies the potential for the continued and additional implementation of online training programs in dental postgraduate training even after the COVID‐19 pandemic. Nevertheless, a causal relationship between distance learning and improved WLB seems to be too superficially. It is necessary to emphasise that the results point to multifactorial correlations that require further research. Moreover, the results have to be seen in a broader context by addressing gender equality issues in dental education like promoting flexible learning formats to support professionals with family commitments. Distance learning might help to reduce gender‐specific barriers to career development.

Further studies are needed to evaluate the long‐term impact of programs on gender equality and professional opportunities.

The study highlights the importance of investigating and integrating the preferences of practicing dentists, particularly considering the growing proportion of female dentists, regardless of the motivation for gender‐ and family‐related training methods. Within university settings and among dental students, there is a noticeable trend towards embracing a ‘back to the future’ concept involving integrating hybrid models that combine digital instruction with practical courses. Several faculties across Germany have either implemented or are contemplating such approaches. Whether this digital shift in dental education will evolve into a lasting and sustainable teaching concept or gradually revert to exclusive face‐to‐face instruction remains to be seen.

Online events became a necessity during the pandemic, even for practicing dentists. Despite the advantages of this type of knowledge acquisition, the often‐missing social component of such events remains a concern. In the future, individuals should be able to choose between training content or personal exchange with colleagues at their discretion. Depending on this, participants can then decide whether to travel or stream from home. Dentists with family responsibilities might benefit from an innovative and creative online offering. Additionally, it is essential that any periods of absence for female dentists—be it due to pregnancy, employment restrictions or childcare commitments—could be partially compensated for utilising digital postgraduate specialisation courses, facilitating their return to the profession.

## Author Contributions

Amely Hartmann: contributed to conception, design, data acquisition and interpretation, drafted and critically revised the manuscript. Sara Steinhoff: contributed to conception, design, data acquisition and interpretation, performed all statistical analyses, drafted and critically revised the manuscript. Bilal Al‐Nawas: contributed to conception, design, data interpretation and critically revised the manuscript. Diana, Heimes: contributed to conception, data acquisition, design and critically revised the manuscript. Peer W. Kämmerer: contributed to conception, design, data acquisition and interpretation, drafted and critically revised the manuscript. All authors gave their final approval and agree to be accountable for all aspects of the work.

## Conflicts of Interest

The authors declare no conflicts of interest.

## Data Availability

Data available on request due to privacy/ethical restrictions.
